# Comparing Biofouling Control Treatments for Use on Aquaculture Nets

**DOI:** 10.3390/ijms151222142

**Published:** 2014-12-02

**Authors:** Geoffrey Swain, Nagahiko Shinjo

**Affiliations:** Center for Corrosion and Biofouling Control, Florida Institute of Technology, Melbourne, FL 32901, USA; E-Mail: ccbc@fit.edu

**Keywords:** net fouling, net coatings, drag, biocides, fouling release, cleaning, test methods

## Abstract

Test panels comprised of uncoated, copper coated and silicone coated 7/8'' (22 mm) mesh knitted nylon net were evaluated to compare their properties and the effectiveness to prevent biofouling. This paper describes test procedures that were developed to quantify the performance in terms of antifouling, cleanability, drag and cost. The copper treatment was the most effective at controlling fouling, however, the silicone treated nets were the easiest to clean. The drag forces on the net were a function of twine diameter, twine roughness and fouling. After immersion, the uncoated nets had the most drag followed by the silicone and copper treatments. The cost of applying silicone to nets is high; however, improved formulations may provide a non-toxic alternative to control fouling.

## 1. Introduction

Biofouling of aquaculture nets causes serious maintenance and operational problems. The direct economic cost to the aquaculture industry of controlling biofouling has been estimated to be 5%–10% of production cost [1]. It has been reported that after only a few months of immersion, biofouling can increase the weight of the netting by as much as two-hundred-fold, and the drag force five-fold [2]. Historically, this additional weight and drag has led to the collapse or failure of several large commercial marine aquaculture structures [3]. Fouling also causes a reduction of the mesh opening and decrease in water circulation through the cage. This results in a significant reduction in carrying capacity and may lead to increased mortalities of the fish [4,5]. Biofouling may also act as a reservoir for parasites and disease and certain fouling species, such as hydroids and anemones, are capable of inflicting harm through nematocysts that will sting and irritate the skin.

At present, net fouling is typically controlled either by changing and cleaning the nets or by the use of chemical antifoulants that contain biocides such as cuprous oxide, copper isothianate, copper pyrithione, zinc pyrithione, zinc oxide econea and others. Whilst the use of untreated nets is safe for the environment, the frequent cleaning and replacement of nets causes stress to the animals, damages the nets, increases maintenance costs and decreases profit margins.

The use of biocides is still the most effective method of controlling fouling, however, there are concerns with respect to the accumulation of biocides in the environment [6]. Some success has been reported with biological control using herbivorous fish or invertebrates to graze biofouling from the net surfaces [7]. Whilst such methods are attractive and may prove helpful, to date there are no published data that have shown it to provide a solution for large-scale operations. Therefore there is still the need to develop better and more environmentally friendly methods to control fouling in the aquaculture industry.

The only contemporary biocide free treatments shown to effectively control fouling are the silicone based fouling release coatings [5,8]. The use of silicones to control fouling was first reported in the early 1970s. Several commercial formulations are now available and regularly being applied to ships and marine structures, however, to date they have not been widely adopted by the aquaculture industry. A recent study by Cassiano *et al.* [9], demonstrated that a commercially available silicone fouling release coating reduced biofouling build up on nets used by the Florida clam industry.

The ideal net treatment would reduce or prevent biofouling, lessen the hydrodynamic drag experienced by the net, be non-toxic to the organisms being cultured, to the consumer and the environment, be easy to apply and be cost effective. This paper presents methods that were developed to quantify the performance of net treatments and presents data from an early study that compared the effectiveness of General Electric RTV 11, a silicone biocide free fouling release coating to Pettit Trinidad^™^, a commercial copper based coating [10]. The general hypothesis was that silicone fouling release coatings will be equal or more effective than biocide based systems at controlling fouling on nets used for marine aquaculture.

The study measured the amount of material required to coat the net, the effectiveness to control fouling, the cleanability of the fouled netting, the hydrodynamic drag characteristics and the costs.

## 2. Results and Discussion

### 2.1. Net Dimensions

The application of the copper coating caused a contraction of the twine and an increase in open area. The application of the silicone coatings increased the twine diameter and reduced the open area of the mesh.

### 2.2. Copper Coating

The application of the copper antifouling caused the net diameter to decrease and the weight to increase. The decrease in twine diameter was due to the volatile component (86% weight percent solids) causing the coating to contract during drying. The area that was occluded by the net therefore decreased (Table 1). The weight of the net increased about four fold due to the weight of cuprous oxide in the coating. Assuming that copper antifouling cost $200.00/gallon and there was 20% waste during application, the additional cost to the net would be about $1.40/ft^2^ (Table 2).

**Table 1:** Average twine diameter and the occluded and open area for the uncoated, copper and silicone coated nets which had a total area 324 in^2^ (0.209 m^2^).

Frames	Twine Diameter (in)	Occluded Area (in^2^)	Open Area (in^2^)	% Net Open Area
Uncoated	0.10	73	251	77%
Copper	0.09	65	259	80%
Silicone	0.15	102	222	69%

### 2.3. Silicone Coating

The application of the RTV11 silicone net treatment caused the twine diameter to increase by about 50%, the open area of the net to decrease and the dry weight of the net to increase almost five fold (Table 2). Assuming that silicone costs $400.00/gallon and there was 20% waste during application, the additional cost to the net would be about $6.40/ft^2^.

**Table 2:** The properties and approximate costs for the 7/8 inch (22.2 mm) net and the application of the silicone and copper coatings.

Property	Net	Copper	Silicone
Weight (lb/ft^2^)	0.027	0.111	0.127
Weight % solids		0.86	1.00
Weight (lbs/gal)		23.45	9.92
% Waste		0.20	0.20
Net treated/gallon (ft^2^)		145.84	62.30
Cost	$35.00/lb	$200.00/gallon	$400.00/gallon
Cost ($/ft^2^)	$0.96	$1.37	$6.42

### 2.4. Fouling

**Figure 1:**
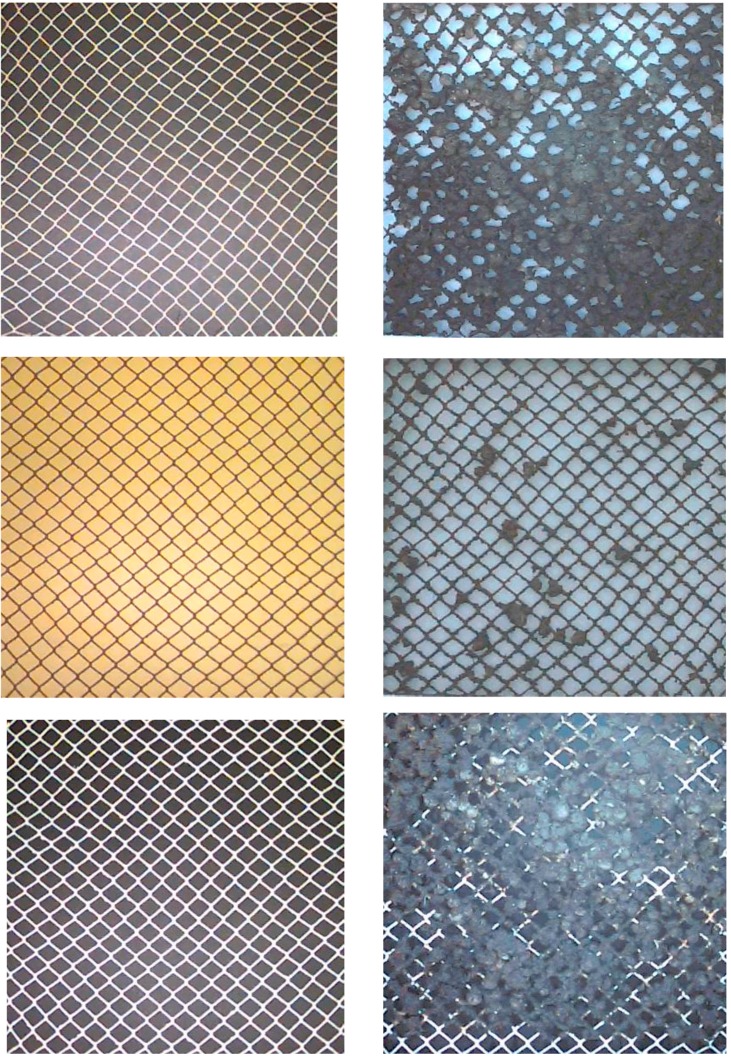
The condition of representative nets at the start of the experiment and after 148 days immersion. Top uncoated; Middle copper; Bottom silicone.

The dominant fouling species found on the nets were silt, slime (bacteria, diatoms and microalgae), and the solitary ascidian, *Styela* sp. (Figure 1). Spatial variations among the samples were observed on all the treatment types. A few small barnacles, *Balanus eburneus*, approximately 1/8'' diameter (3.1 mm), were observed on some of the nets for all treatment types and they were mainly attached at the nodes of the nets. Encrusting bryzoans were observed on the silicone and untreated nets wrapped around the twine. Although encrusting bryzoans did not significantly contribute to the occluded area, they provided attachment points for other organisms. A large amount of hydrozoans about 1/8'' length (3.1 mm) were observed mainly on the untreated nets. Solitary ascidians were the major fouling organism and contributor to blocking the mesh openings. Their size ranged from 1/8'' to 3/4'' (3.1 to 19 mm) diameter, and they were occasionally piled up on each other. Ascidians also added weight and about 1'' (25.4 mm) thickness to the samples. The diversity of the fouling community appeared to be greatest on the untreated and silicone nets.

**Figure 2:**
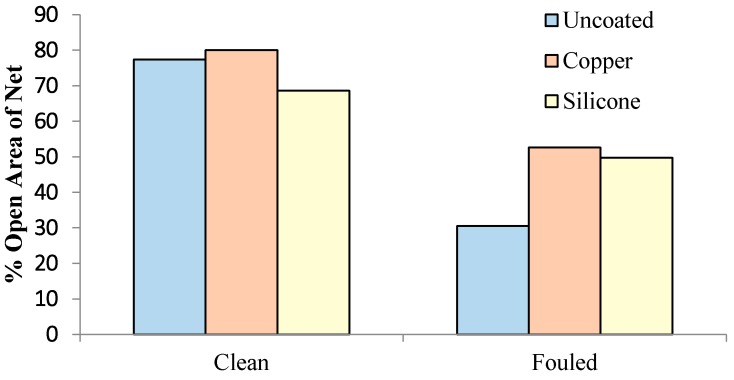
Average percent area of net open.

Fouling greatly reduced mesh openings on untreated samples while the copper treated nets had the least fouling (Figure 1 and Figure 2). The mesh opening was reduced by 28% for the untreated nets, by 10% for the copper and by 12% for the silicone. The silicone treated nets were fouled less than untreated nets but more than copper treated nets. The diversity of the fouling community on the silicone was similar to untreated nets and included *Styela* sp., *Balanus eburneus* and encrusting bryozoans. The copper treated nets had the least amount of fouling, and the fouling species were dominated by silt, slime, and algae with only a few *Styela* sp.

### 2.5. Cleanability

**Figure 3:**
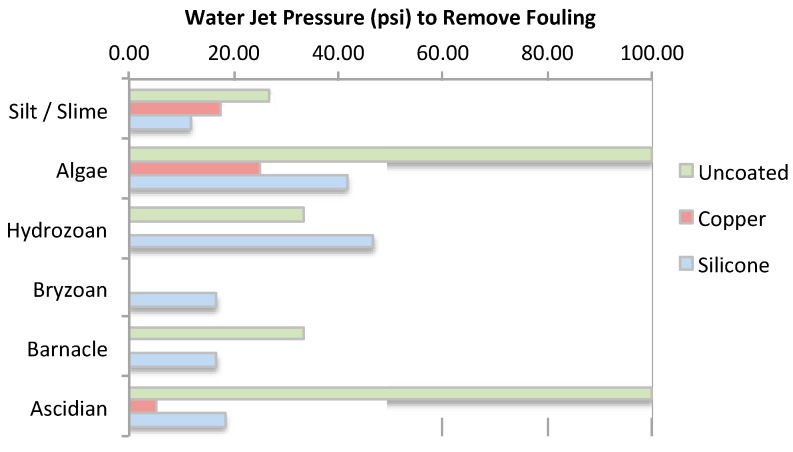
Average water-jet pressure required to remove the major fouling categories from the different net treatments.

**Figure 4:**
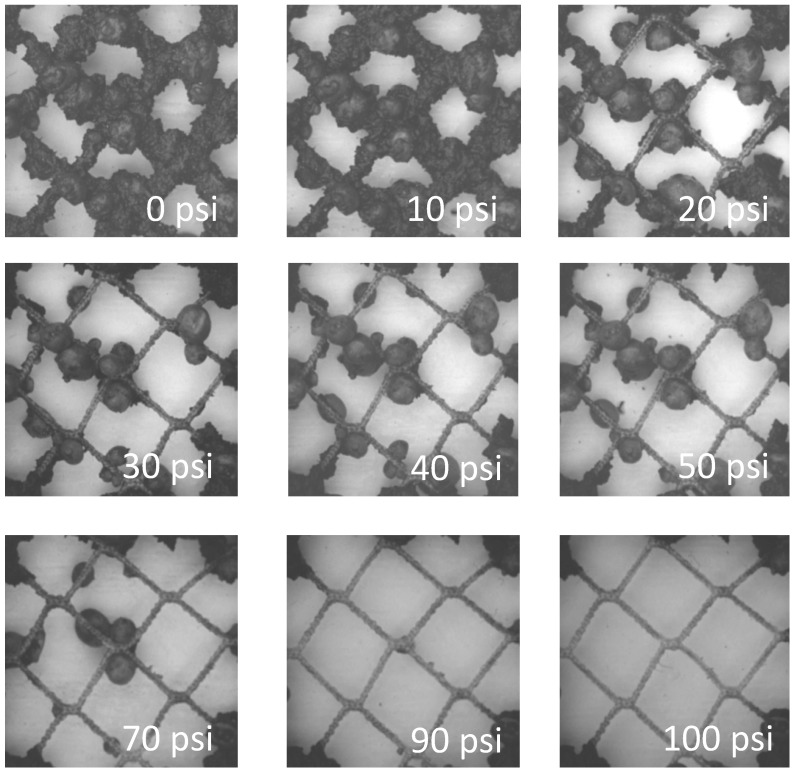
Fouling removal from the uncoated net using the water-jet.

The water-jet pressure required to remove fouling differed between the net treatments (Figure 3 and Figure 4). The untreated nets required the highest pressure for fouling removal and the copper coated nets the lowest pressure. Algae and ascidians were firmly attached to the untreated net. Other fouling was easily removed by 20 to 40 psi (0.138 to 0.276 MPa) water jet pressures.

Fouling removal from the untreated nets increased as the water-jet pressure increased from 10 to 100 psi (0.0689 to 0.689 MPa), however, the nets did not clean back completely and stains remained on twine even when the maximum pressure was applied. Some ascidians, algae, and hydrozoans remained at over 100 psi (0.689 MPa) on untreated nets. Fouling was removed from the copper coated nets at about 30 psi (0.207 MPa). Due to the toxicity of copper, the nets were less fouled and lacked the large numbers of ascidians and barnacles which made cleaning easier.

The majority of fouling was removed from the silicone treated net at below 30 psi (0.207 MPa). This was mainly due to removal of ascidians and small barnacles located at the nodes of the nets, which were also easily removed at around 20 psi (0.138 MPa). Most of the algae were removed at less than 20 psi (0.138 MPa), however, the algae attachment bases (black dots) remained even at higher pressures. Although hydrozoans were detached or loosened at less than 20 psi (0.138 MPa), they remained on the net since they wrapped around the twine.

### 2.6. Hydrodynamic Performance

**Figure 5:**
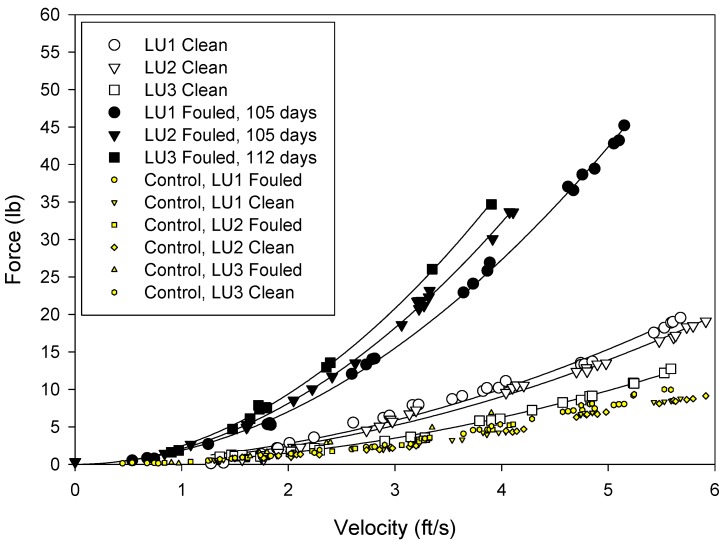
Drag forces measured on the uncoated nets. White filled symbols represent clean nets and black filled symbols represent fouled nets. Shaded points without regression lines are the force readings on the control force gage (blank frame).

**Figure 6:**
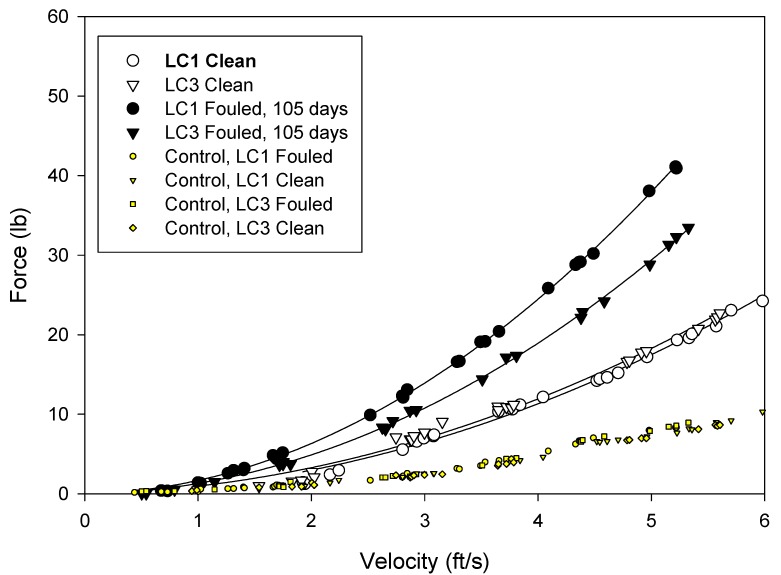
Drag forces measured on the copper coated nets. White filled symbols represent clean nets and black filled symbols represent fouled nets. Shaded points without regression lines are the force readings on the control force gage (blank frame).

**Figure 7:**
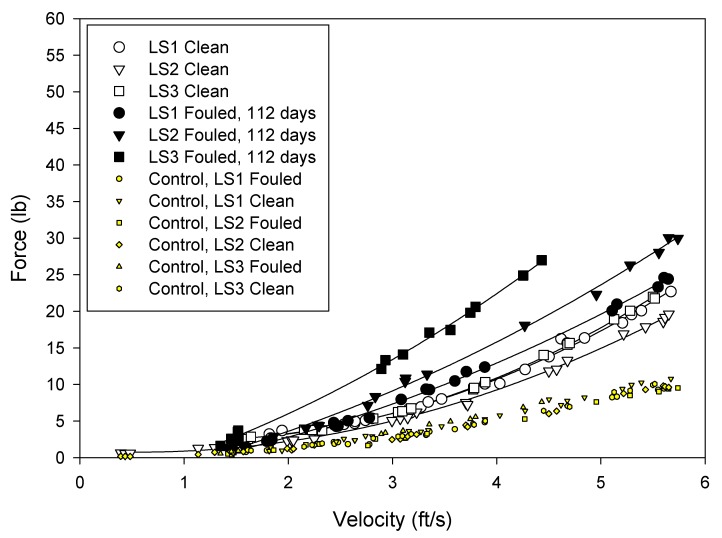
Drag forces measured on the silicone coated nets. White filled symbols represent clean nets and black filled symbols represent fouled nets. Shaded points without regression lines are the force readings on the control force gage (blank frame).

The drag forces were measured at seawater velocities from 1 to 5 ft/s (0–1.5 m/s) (Figure 5, Figure 6 and Figure 7). The force acting on the frame was subtracted from the values measured for the test nets and so the values reflect the net drag only.

The analyses of the net hydrodynamics were performed with respect to measured force, projected area and drag coefficient. The copper coated nets presented the smallest surface area to the flow, however, the coating created a roughness that added to the drag. Whilst the silicone coatings had increased twine diameter, it formed a smooth surface and this lessened the drag on the net. The hydrodynamic performance of the nets was evaluated in terms of drag coefficient (Table 3) using the using the following equation.

(1) $$C_{\text{d}}=F/(0.5\times \text{ρ}\times A\times V^{2})$$

where:

*C*_d_ = drag coefficient
*F* = measured force (lbs)
ρ = density of seawater (1.9848 slugs/ft^3^)
*A* = surface area of net (ft^2^)
*V* = Velocity (ft/s)

**Table 3:** Average drag forces measured on the nets in the clean and fouled condition and the calculated drag coefficient at 5 ft/s (1.5 m/s) for the clean nets.

Treatment	1 ft/s		5 ft/s		*C*_d_
	Clean	Fouled	Clean	Fouled	Clean at 5 ft/s
Uncoated	0.61	2.12	13.05	49.62	1.04
Copper	0.86	1.45	17.88	33.63	1.60
Silicone	0.60	1.16	16.70	25.79	0.95

The drag coefficients provide a measure of the “roughness” of the net. For the treatments examined in these tests, the silicone coated nets had the lowest drag coefficient and the copper coated nets the highest.

After 148 days static immersion, all the nets became fouled. The uncoated nets had the highest fouling and drag. This was followed by the copper and the silicone coated nets.

The data generated by these tests demonstrated that drag, and therefore water circulation, in unfouled net cages will be a function of the total twine area and roughness. As the nets become fouled the drag forces increase. Drag was greatest on the untreated fouled nets and least on the fouled silicone treated nets.

Previous studies [2,11] have indicated that hydrodynamic drag on nets depends not only on physical dimensions but also on texture and net material. This was also observed in these experiments, and it appeared that the surface roughness associated with uncoated and copper coated nets increase the viscous drag on the net. Furthermore, it was observed that the net deformation was greatest on untreated nets, followed by silicone and copper treatments. This was a function of net modulus. Copper coated nets had the highest modulus, followed by the silicone coated net and uncoated net. Distortion of the low modulus nets may have reduced the angle of attack and consequently resulted in reduced drag [12,13]. Deformation was visually observed as “blooming” of the net samples while under flow and was especially pronounced at higher velocities.

## 3. Method

The method was designed to enable the exposure of different net treatments to fouling, to measure the effectiveness of the treatments to control fouling and to quantify the effect of the net condition on resistance to water flow. Knotless 7/8'' (22 mm) mesh, #252, 75lb (334 N) test nylon net was selected as the test material. Samples consisted of three replicates of three treatments: untreated, copper antifouling paint, and a silicone fouling release coating. The net material was cut to a 20'' × 20'' square. The copper coating chosen for these tests was Pettit Trinidad™ (Kop-Coat Marine Group, Rockaway, NJ, USA). This is a commercially available biocide based antifouling paint with about 86% weight percent solids. It functions by the release of copper ions at a rate that prevents the settlement and growth of organisms. It was applied to the net by brush. The silicone fouling release coating chosen for this study was General Electric RTV11™ (General Electric, Schenectady, NY, USA), a 100% solids mold release silicone. This was used as one of the base silicones for a fouling release research project undertaken by General Electric and funded by the Environmental Security Certification Program and the Defense Advanced Research Project Agency [14,15]. The silicone fouling release coatings function by creating a low modulus, low surface energy and smooth surface that reduces the adhesion strength of marine organisms that are recruited to the surface [8,16]. It was applied by a two-dip process, which insured that the twine was totally encased in a smooth coating. The amount of coating and increase in weight of the nets were measured during each coating application.

**Figure 8:**
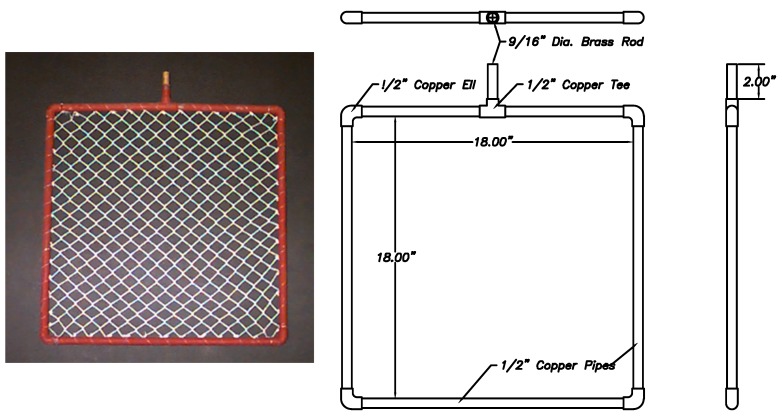
Test frame.

The nets were trimmed and attached to 18'' × 18'' (0.46 m × 0.46 m) frames constructed from half inch diameter copper pipe using 20lb test monofilament nylon fishing line (Figure 8). Copper pipes were selected for the sample frame material because of their rigidity and corrosion resistance. The frames were coated with copper anti-fouling paint for protection against fouling and to ensure smooth surfaces. After construction, the nets were photographed and the Sigma Scan™ (version 3.0, SPSS software, IBM, Armonk, NY, USA) image analysis program was used to measure the net area. The net dimensions were measured using high resolution close-up 3'' × 3'' (7.6 mm × 7.6 mm) images of the samples (Table 1).

The nets were subjected to biofouling at the Florida Institute of Technology static immersion site located in the Indian River Lagoon on the east coast of Florida from 20 June 1996 to 15 November 1996, a period of 148 days. At the end of the exposure period a visual estimate of the fouling types present on each net was made. Digital images were taken and the Sigma Scan™ image analysis program was used to measure the area of the net that was occluded by the fouling and the area of the net that was open and free of fouling. The copper frames were then lightly cleaned using a towel to remove biofilms and insure that their surface condition was the same as at the start of the tests. The nets were then tested for hydrodynamic drag.

**Figure 9:**
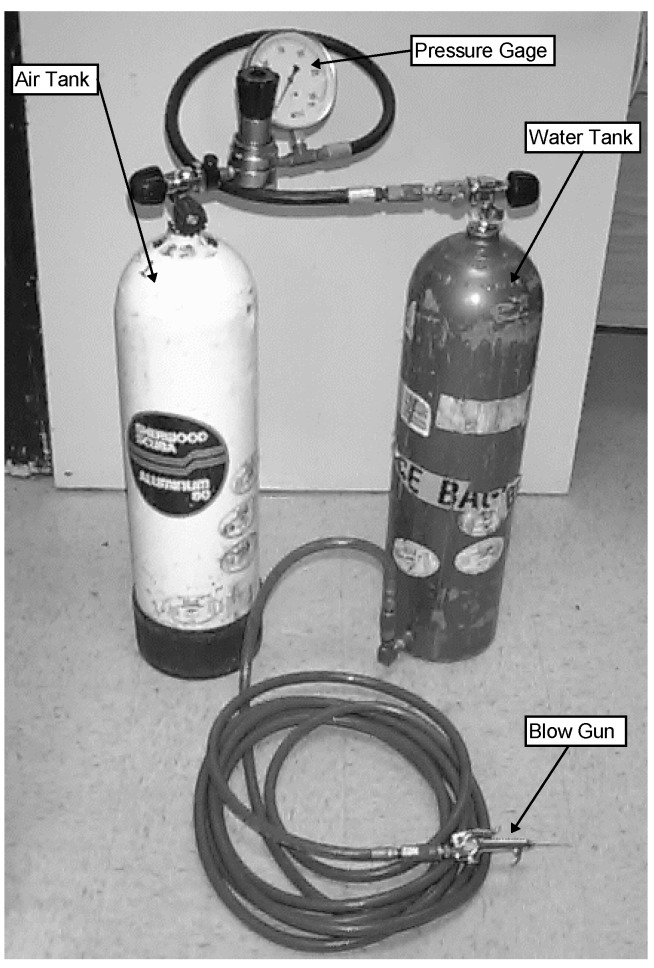
Water-jet method.

The cleanability of fouling from nets was measured using a water-jet apparatus [17,18]. The system consists of two SCUBA tanks a pressure regulator, and a quick connect air gun with a 1/16'' (1.6 mm) diameter nozzle (Figure 9). The first SCUBA tank contained compressed air and the pressure regulator controlled pressurized air to the second SCUBA tank which contained water. The air pressure was adjusted by the regulator between 0 to 100 psi at 10 psi increments (0 to 0.689 MPa at 0.069 MPa increments). A section of the fouled net was selected and photographed. The air pressure was set at 10 psi and the water jet applied to the area until the maximum amount of fouling that could be removed at this setting was achieved. The area was photographed, the pressure increased by 10 psi and the process repeated until all the fouling was removed or the maximum pressure (100 psi) was reached.

**Figure 10:**
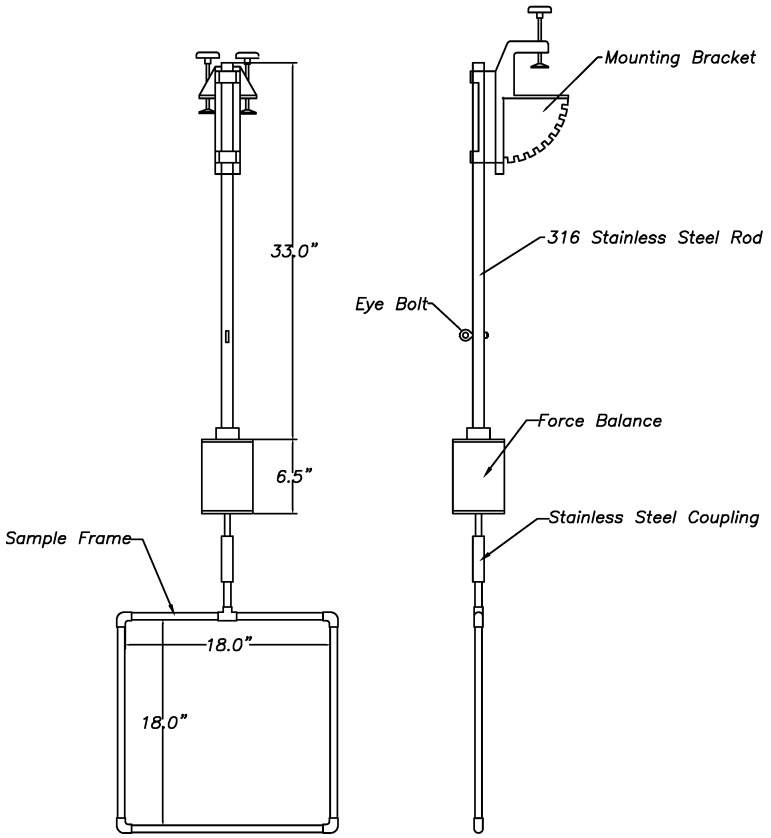
Force balance assembly.

The drag forces acting on the nets were measured by attaching the frames to load cells (Figure 10) that were mounted on either side of an aluminum cross member that was placed in the center of an 24 foot (7.3 m) research boat and subjecting them to increasing seawater velocities. The experimental nets mounted in their frames were attached to one shear beam load cell and a frame with no net was attached to the second shear beam load cell as a control. The frames were orientated perpendicular to the flow at a depth of 20 inches (0.5 m) and the boat was run at increasing speeds from 0–5 ft/s (0–1.5 m/s). The water velocity at the net was measured using a United Sensor pitot static pressure probe PAE-8-M-W, (United Sensor Corporation, Amherst, NH, USA) and an Omega wet-wet differential pressure transducer PX26 0–5 psi, (Omega Engineering, Inc., Stamford, CT, USA). The drag forces were measured from the load cells. Data were sampled at 100 Hz for 10 s at each speed.

## 4. Conclusions

An understanding and comparison of the performance of different net treatments to control fouling is important to help identify the most effective and environmentally sustainable method for a particular application. This paper described test procedures that were developed to quantify the performance in terms of antifouling, cleanability, drag and cost. The net treatments used in this study have since been superseded by several new biocide based and improved silicone fouling release formulations, however, the basic properties, mechanisms and costs of the new treatments are similar.

The study measured a five-fold increase in drag due to fouling on the uncoated nets after about 4 months immersion. Similar effects have been reported in the literature [2] and reinforce the need for an economical and environmentally acceptable biofouling control method. The copper treatment, used in this study, reduced fouling and caused a reduction in twine diameter, however, the coating roughness caused a significant increase in the drag coefficient. The silicone coatings fouled, however, fouling removal was easier than on untreated nets and the smoothness of the coating decreased the drag coefficient. The cost of the copper and silicone increased the raw material costs of the net by about 1.5 and 6.0 times respectively. The question remains as to whether the high costs of a silicone treated net can be justified in terms of improved biofouling control and water quality for the fish, reduced maintenance and increased life expectancy of the nets, and the removal of biocides from the environment.
